# Azvudine, A Novel Nucleoside Reverse Transcriptase Inhibitor Showed Good Drug Combination Features and Better Inhibition on Drug-Resistant Strains than Lamivudine *In Vitro*

**DOI:** 10.1371/journal.pone.0105617

**Published:** 2014-08-21

**Authors:** Rui-Rui Wang, Qing-Hua Yang, Rong-Hua Luo, You-Mei Peng, Shao-Xing Dai, Xing-Jie Zhang, Huan Chen, Xue-Qing Cui, Ya-Juan Liu, Jing-Fei Huang, Jun-Biao Chang, Yong-Tang Zheng

**Affiliations:** 1 Key Laboratory of Animal Models and Human Disease Mechanisms of Chinese Academy of Sciences & Yunnan Province, Kunming Institute of Zoology, Chinese Academy of Sciences, Kunming, Yunnan, China; 2 College of Chemistry and Molecular Engineering, Zhengzhou University, Zhengzhou, Henan, China; 3 Kunming College of Life Science, University of Chinese Academy of Sciences, Kunming, Yunnan, China; 4 State Key Laboratory of Genetic Resources and Evolution, Kunming Institute of Zoology, Chinese Academy of Sciences, Kunming, Yunnan, China; 5 College of Pharmacy and Chemistry, Dali University, Dali, Yunnan, China; Institut Pasteur of Shanghai, Chinese Academy of Sciences, China

## Abstract

Azvudine is a novel nucleoside reverse transcriptase inhibitor with antiviral activity on human immunodeficiency virus, hepatitis B virus and hepatitis C virus. Here we reported the *in vitro* activity of azvudine against HIV-1 and HIV-2 when used alone or in combination with other antiretroviral drugs and its drug resistance features. Azvudine exerted highly potent inhibition on HIV-1 (EC_50_s ranging from 0.03 to 6.92 nM) and HIV-2 (EC_50_s ranging from 0.018 to 0.025 nM). It also showed synergism in combination with six approved anti-HIV drugs on both C8166 and PBMC. In combination assay, the concentrations of azvudine used were 1000 or 500 fold lower than other drugs. Azvudine also showed potent inhibition on NRTI-resistant strains (L74V and T69N). Although M184V caused 250 fold reduction in susceptibility, azvudine remained active at nanomolar range. In *in vitro* induced resistant assay, the frequency of M184I mutation increased with induction time which suggests M184I as the key mutation in azvudine treatment. As control, lamivudine treatment resulted in a higher frequency of M184I/V given the same induction time and higher occurrence of M184V was found. Molecular modeling analysis suggests that steric hindrance is more pronounced in mutant M184I than M184V due to the azido group of azvudine. The present data demonstrates the potential of azvudine as a complementary drug to current anti-HIV drugs. M184I should be the key mutation, however, azvudine still remains active on HIV-1_LAI-M184V_ at nanomolar range.

## Introduction

Nucleoside reverse transcriptase inhibitors (NRTIs) were the first class of compounds to be used in anti-human immunodeficiency virus (HIV)-1 therapy and are essential components in highly active antiretroviral therapy (HARRT) [1]. All clinical NRTIs belong to the family of 2′, 3′-dideoxynucleoside (ddNs) [2]. These compounds become active after being phosphorylated into NRTI triphosphate derivatives (NRTI-TPs) and compete with endogenous deoxynucleoside triphosphates (dNTPs) for incorporation into the primer strand by reverse transcriptase (RT). Since NRTI-TPs do not have a 3′-OH group on the sugar or pseudosugar moiety, NRTI monophosphate (NRTI-MP) incorporated into primer strand prevents further elongation [3]. Despite many inhibitors of HIV-1 RT are available showing good clinical effectiveness in combination regimens, long-term usage often results in the development of viral resistance or long-term toxicity. Thus it is necessary to identify new agents with higher efficacy against the drug-resistant HIV-1 strains.

HIV-1 mutants resistant to ddNs can distinguish between ddN and physiologic 2′-deoxynucleoside (dN). They exclude ddN from the active center of RT and selectively remove the incorporated ddN from proviral DNA terminus. Therefore, nucleoside drugs that can prevent the emergence of drug-resistant HIV variants must have a 3′-OH as the chain terminator of proviral DNA biosynthesis. Wang et al has reported that the 4′-*C*-substituted-2′-deoxy-nucleosides (4′sdNs) could prevent the emergence of drug-resistant HIV variants [4]. The 4′sdNs have all functional groups of dNs to prevent viral discrimination whereas the 3′-OH of 4′sdNs is not available for proviral DNA biosynthesis. Thus, 4′sdNs can act as the chain terminator of proviral DNA biosynthesis and active against both HIV and ddN-resistant HIV strains. Furthermore, modifications of ribofuranosyl moiety of nucleosides with functional groups, such as azido, cyano, and ethynyl at the 4′-position can affect the nucleoside’s electronic properties and conformational shape, leading to improved activity [5].

**Figure 1:**
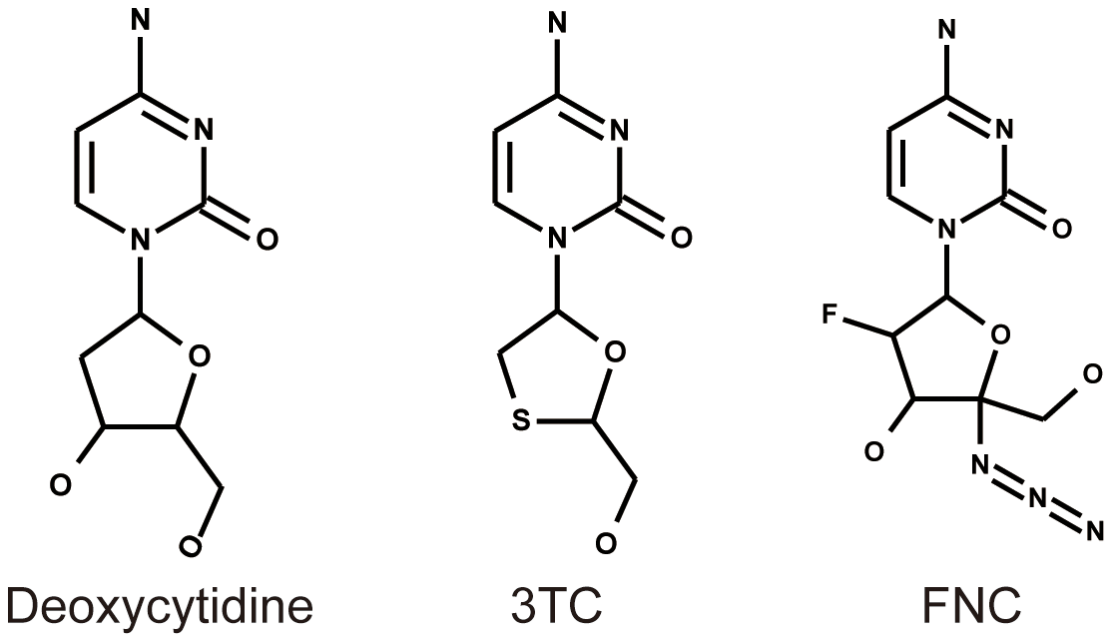
Chemical structures of FNC and 3TC.

Among these 4′sdNs, 2′-deoxy-2′-β-fluoro-4′-azidocytidine, also known as azvudine or FNC, is a novel cystidine ananlogue (Fig. 1) that is an excellent substrate for deoxycytidine kinase and can be phosphorylated more efficiently than deoxycytidine [6], [7]. Azvudine (FNC) retains 3′-OH group as the chain terminator of proviral DNA biosynthesis. Previous studies have demonstrated the antiviral activities of FNC on HIV, hepatitis B virus (HBV) and hepatitis C virus (HCV) [8]–[10]. Since HIV and HBV share common transmission routes, about one-tenth of HIV-infected patients are co-infected with chronic hepatitis B [11]. It implies that FNC may not only be developed into anti-HIV drug, like lamifudine (3TC) and Emtricitabine (FTC), but also into anti-HBV drug.

3TC is the most common nucleoside analogues used in first-line combination therapy for treating HIV-1 infections but drug resistance has been detected in both cell culture and infected patients [11], [12]. 3TC treatment of HIV-1 infected patients selects for drug-resistant variants with sequential mutation at position 184 from Met to Ile, followed by Ile to Val. The M184V substitution is associated with high-level resistance to 3TC and K65R mutation confers intermediate to high-level resistance to the 3TC [13], [14]. Thus, the development of novel compounds that are active against drug-resistant HIV-1 to prevent or delay the emergence of resistant HIV-1 variants is urgently needed [15].

Azvudine (FNC) is a cystidine analogue similar as 3TC except with a 3′-OH. Theoretically,FNC will show better inhibitory effect to drug-resistant HIV variants than 3TC. Though the hydrochloride salt of FNC did show good suppression to NRTI-resistant viral strains, the antiviral activity decreased upon M184V mutation of HIV-1 [4]. In our present study, we first compared the anti-HIV activities of FNC and 3TC in parallel assays. The resistant mutations selection of FNC *in vitro* was using dose escalation methods to predict drug resistance of FNC. Furthermore, we evaluated clinical application of FNC by combination assay with different target approved drugs.

## Materials and Methods

### Ethics statement

Ethical approval for the study and the informed consent process were approved by the Ethics Committee of Kunming Institute of Zoology, Chinese Academy of Sciences (Approval Number: SWYX-2006011, 2011016). Written informed consent was obtained from all participants prior to the study. The study was conducted in accordance with basic principles of the Helsinki declaration and the relevant international rules.

### Compounds and reagents

FNC with a purity of 98.5% and 3TC with a purity of 99.6%, were synthesized by Dr. Jun-biao Chang, Zhengzhou University, China. 3-(4,5-dimethyl-2-thiazolyl)-2,5-diphenyl-2H-tetrazolium bromide (MTT), sodium dodecyl sulfate (SDS), N, N-dimethylformamide (DMF), phytohemagglutinin (PHA-p), interleukin-2 (IL-2), Fc-specific anti-mouse IgG, horseradish peroxidase (HRP)-labeled goat anti-rabbit IgG and zidovudine (AZT) were purchased from Sigma-Aldrich company. Raltegravir (RAL) was purchased from Selleck Chemicals. Nevirapine (NVP) was purchased from US Pharmacopeia. Enfuvirtide (T-20) was purchased from Roche Inc. RPMI-1640 media and fetal bovine serum (FBS) were purchased from Invitrogen, USA. FBS was heated at 56°C, 30 min to inactivate complements. Indinavir (IDV) was kindly gifted by Dr. Yu-Ye Li. Ficoll-Hypaque was purchased from Haoyang Biotechnonlogy Inc. Rabbit anti-p24 polyclonal antibody and mouse anti-p24 monoclonal antibody were prepared in our laboratory. Custom primers and fluorophore-labeled probes were synthesized by Invitrogen.

### Cells and viruses

C8166 cells were kindly donated by the Medical Research Council (MRC), the AIDS Reagent Project, U.K, and were maintained in RPMI-1640 with 10% FBS in humidified incubator with 5% CO_2_ at 37°C [16]. Peripheral blood mononuclear cells (PBMCs) were isolated from healthy donors by Ficoll-Hypaque density gradient centrifugation as described by the manufacturer’s instructions (Ethical Approval Number: SWYX-2011016). PBMCs were stimulated with 10% FBS, 5 µg/ml PHA and 50 U/ml IL-2 for three days before experiments.

Laboratory adaptive strains, including HIV-1_IIIB_, HIV-1_RF_, HIV-1 reverse transcriptase (RT) resistant strain, HIV-1_LAI-M184V_ and HIV-1_L74V_, fusion inhibitor resistant strain pNL4-3_gp41 (36G) V38A/N42T_, HIV proteaseGene Mutants HIV-1_RF/V82F/184V_ and HIV-1_L10R/M46I/L63P/V82T/I84V_ were kindly donated by NIH. Clinical isolated strains, including HIV-1_KM018_, HIV-1_TC-1_ and HIV-1_WAN_ were isolated from local AIDS patients (Ethical Approval Number: SWYX-2006011) and were propagated by co-culture with healthy PBMCs. All virus stocks were stored in small aliquots at −70°C.

### Cytotoxicity assays

Cytotoxicity was assayed by MTT colorimetric reduction as previously described [17], [18]. Briefly, a serial concentration of FNC was added to a 96-well plate, followed by 100 µl 4×10^4^ C8166 cells (5×10^5^ cells for PBMC). After incubation at 37°C, 5% CO_2_ for 3 days (7 days for PBMCs), 20 µl MTT was added each well. After incubation for 4 hours, 100 µl supernatant was removed and 100 µl 20%SDS-50%DMF was added. The plate was incubated at 37°C overnight. The optical absorbance was measured by ELISA reader (ELx800, Bio-Tek, VT, USA) at 570 nm and 630 nm, and 50% cytotoxicity concentration (CC_50_) was calculated. 3TC and AZT were used as control.

### Anti-HIV activity *in vitro*

C8166 cells were infected with different HIV-1 or HIV-2 laboratory strains and resistant strains at different serial concentration compounds with multiplicity of infection (MOI) of 0.075∼0.6. PHA-stimulated PBMCs were incubated with different clinical strains in RPMI-1640 (with 10% FBS, 50 U/ml IL-2 and 2 µg/ml polybrene) at MOI of 0.1. After infection at 37°C in 5% CO_2_ for 2 hours, C8166 cells were washed three times to remove free viruses and re-suspended by RPMI-1640 (with 10% FBS). 100 µl 4×10^4^ cells (5×10^5^ cells for PBMC) were seeded each well in a 96-well plate with gradient concentration of FNC. The plate was placed in a humidified incubator at 37°C, 5% CO_2_. 3TC and AZT were used as control. After incubation of 3–7 days, the percentage inhibition of syncytia formation was scored or the level of p24 was measured by ELISA [19] and 50% effective concentration (EC_50_) were calculated.

### Combination antiviral activity assay

Antiviral effects of FNC in combination with non-nucleoside reverse transcriptase inhibitor (NNRTI)- nevirapine (NVP), nucleoside reverse transcriptase inhibitors (NRTI)- zidovudine (AZT) and 3TC, fusion inhibitor (FI)- enfuvirtide(ENF), integrase inhibitor (INI)- reltegravir (RAL) and protease inhibitor (PI)- indinavir (IDV) were tested on HIV-1_IIIB_ infected C8166 cells and HIV-1_TC-1_ infected PBMC as previous report [20]. The syncytia formation of C8166 was determined under microscope on day 3 and the p24 antigen level in supernatant of PBMC was tested by ELISA on day 10. The combination index (CI) was calculated according to Chou-Talalay Method [21] using Calcusyn software (Biosoft, USA). A drug combination was defined according to CI, as follows: <0.1, very strong synergism; 0.1–0.3, strong synergism; 0.3–0.7, synergism; 0.7–0.85, moderate synergism; 0.85–0.90, slight synergism; 0.90–1.10, nearly additive; 1.10–1.20, slight antagonism; 1.20–1.45, moderate antagonism; 1.45–3.3, antagonism; 3.3–10, strong antagonism; >10, very strong antagonism. Combination volume values were calculated at their 50% confidence level.

### Quantification of HIV-1 DNA species

Real-time quantitative PCR was performed to determine the level of HIV-1 DNA in cells. Briefly, C8166 cells (10^6^ cells/mL) were seeded into a clear 24-well plate and infected with HIV-1_IIIB_ (MOI = 0.1) in the absence or presence of FNC (2 nM) or 3TC (2 µM). After incubation at 4°C for 2 hours, the infected cells were transferred to 37°C and then cellular DNA was extracted at different time-points (0, 1, 2, 4, 6, and 8 h) for real-time quantitative PCR assay. The strong-stop minus-strand DNA (ssDNA) and full-length double-stranded DNA (late-RT) were detected. The primers for real-time PCR were as follows: ssDNA forward, 5′-GCCTCAATAAAGCTTGCCTTGA-3′; ssDNA reverse, 5′-TGACTAAAAGGGTCTGAGGGATCT-3′; ssDNA probe, 5′-FAM-AGAGTCACACAACAGACGGGCACACACTA-TAMRA-3′; late RT forward, 5′-TGTGTGCCCGTCTGTTGTGT-3′; late RT reverse, 5′-GAGTCCTGCGTCGAGAGAGC-3′; late RT probe, 5′-(FAM)-CAGTGGCGCCCGAACAGGGA-(TAMRA)-3′ [22]. After initial incubation at 95°C for 2 min, 40 cycles of amplification were carried out at 15 sec. at 95°C followed by 30 sec. at 60°C. Standard curves for quantification of the late RT amplicon were prepared by serial dilution of matching cloned DNAs of known concentrations.

### Selection of drug-resistant HIV-1 variants

FNC and 3TC-resistant viruses were selected by adding the compounds progressively in HIV-1_IIIB_ infected C8166 cells according to previously described methods with some modifications [23]. Briefly, 1×10^6^ of HIV-1_IIIB_ infected C8166 cells (MOI = 0.075) were cultured in a 6-well plate in presence of 500 pM FNC or 1 µM 3TC (the inhibition of HIV >99% at these concentrations). The medium was changed twice a week and cultures were examined under microscope after each replacement. Once treated cells attained syncytia formation over 80%, supernatants were collected and stored in aliquots at −70°C. Viral p24 antigen was determined by ELISA as described previously. Collected viruses were used for next infection and the newly-infected C8166 cells were treated by FNC or 3TC with the concentration doubled. The process was repeated until the concentrations reached 32 nM for FNC and 64 µM for 3TC at passage 21(P-21). HIV-1_IIIB_ infected C8166 cells of the same passages without treatment were used as negative control. The virus aliquots collected at P-5, P-11, P-16 and P-21 were used for genotypic assay and the viruses of P-21 were used for phenotypic assay.

### Phenotypic analysis of resistant variants

Phenotypic analysis of C8166 cells was performed as described previously [24]. Briefly, the C8166 cells were infected with the FNC- and 3TC-selected HIV-1_IIIB_ at P-21 (MOI = 0.075) and then treated with a serial dilution of FNC or 3TC in triplicate. After incubation of 72 hours, supernatant was tested by ELISA. The 50% effective concentrations (EC_50_) of FNC and 3TC were determined. The resistance of virus variant was expressed as the fold change (FC) of EC_50_. The susceptibility fold-change of virus variants was defined as the ratio of EC_50_ of mutated drug resistant variants to that of wild HIV-1_IIIB_.

### Genotypic resistance assay

Viral RNA of selected resistant strain was extracted from viral stocks using High Pure Viral RNA Kit (Roche, Mannheim, Germany). cDNA was synthesized by reverse transcriptase kit (TAKARA) and amplified by PCR as described before [24]. The primers were 5′- CCCATTAGCCCTATTGAGACTGT-3′ and 5′- TAGTACTTTCCTGATTCCAGCAC-3′. The PCR reaction consisted of an initial denaturation at 94°C for 2 min, followed by 35 amplification cycles (94°C for 30 s, 60°C for 30 s, 72°C for 2 min) and final extension at 72°C for 10 min. Full-length HIV RT gene of 1680 bp was produced and ligated into pCR2.1 T-vector (Invitrogen). 253 positive clones were sequenced by Invitrogen Inc. The full RT sequence derived from isolated viral mutant and control virus was aligned with wild type HIV-1_IIIB_ strain and amino acid mutations were identified with MEGA 5.1 software.

### Predicting the binding mode of FNC and 3TC to RT

The binding mode of compounds FNC and 3TC to RT of wild type and mutant forms (M184V/I) was predicted by the program POSIT of OpenEye (http://www.eyesopen.com/). POSIT is designed to use bound ligand information to improve pose prediction [25]. The complex structure of RT with Zidovudine-TP (AZTTP) (PDB 3V4I) was obtained from the Protein Data Bank and was prepared by removing solvent molecules and adding hydrogen using Discovery Studio 3.1 (http://accelrys.com/products/discovery-studio/). Furthermore, the structures of RT with mutations (M184V/I) were also modeled using Discovery Studio 3.1. FNC and 3TC are taken up by cells and converted into FNCTP and 3TCTP. The structures of compounds FNCTP, 3TCTP and Deoxycytidine-TP (DCTP) were downloaded from PubChem [26], whose CID numbers were 49771639, 454110 and 65091, respectively. DCTP is a substrate of RT and used as a control. To predict the binding model, these compounds were docked into the active site of RT by POSIT using the pose of AZTTP in RT as a guide. All parameters were set to the default values. The three-dimensional structures of the docked complex were visualized using Pymol [27]. TM-align was used for structural alignment [28].

## Results

### Anti-HIV activity of FNC

To evaluate the antiviral activity of FNC, the lab-adaptive strains (IIIB, RF) and clinical strains (KM018, TC-1, WAN) were used to infect HIV sensitive C8166 cells or PHA-stimulated PBMCs and the commercially available NRTI-3TC was used as control. As shown in Table 1 and Table 2, FNC displayed strong inhibition on wild-type HIV-1_IIIB_ and HIV-1_RF_ with the 50% effective concentration values (EC_50_) ranging from 30 to 110 pM. FNC was about 1835- to 11671-fold more potent than 3TC.

**Table 1:** Anti-HIV-1 activities of FNC, 3TC and AZT in cell cultures^a^.

Compound	Cells	Virus	Subtype	CC_50_s (µM)^b^	EC_50_s (nM)
FNC	C8166	HIV-1_RF_	B	2380±50	0.03±0.02
		HIV-1_74V_	B		0.11±0.01
		HIV-1_L10R/M46I/L63P/V82T/I84V_	B		0.14±0.01
		HIV-1_RF V82F/184V_	B		0.37±0.02
		pNL4-3_ gp41 (36G) V38A/N42T_	B		0.36±0.03
		HIV-2_ROD_	A		0.018±0.006
		HIV-2_CBL-20_	A		0.025±0.002
	PBMC	HIV-1_KM018_	B/C**^c^**	3.07±0.23	6.92±0.59
		HIV-1_TC-1_	CRF01AE		0.34±0.04
		HIV-1_WAN T69N_	CRF01AE		0.45±0.09
3TC	C8166	HIV-1_RF_	B	930±190	350.13±206.48
		HIV-1_74V_	B		243.13±10.10
		HIV-1_ L10R/M46I/L63P/V82T/I84V_	B		415.59±11.85
		HIV-1_RF V82F/184V_	B		>1000
		pNL4-3_ gp41 (36G) V38A/N42T_	B		385.66±36.24
		HIV-2_ROD_	A		256.66±43.63
		HIV-2_CBL-20_	A		319.36±29.83
	PBMC	HIV-1_KM018_	B/C**^c^**	825.98±30.52	339.88±12.18
		HIV-1_TC-1_	CRF01AE		51.72±3.08
		HIV-1_WAN T69N_	CRF01AE		86.53±11.02
AZT	C8166	HIV-1_RF_	B	5090±260	ND
	PBMC	HIV-1_KM018_	B/C**^c^**	845.31±66.23	478.35±45.50

***a*** All data represent means± standard deviation for three separate experiments.

***b*** CC_50_, 50% cytotoxic concentration.

***c*** B/C, B/C recombinant.

**Table 2:** Anti-HIV-1 activities of FNC, 3TC and AZT against RT-resistant strains in C8166 cells^a^.

HIV strain	FNC		3TC		AZT	
	EC_50_ (nM)	FC^b^	EC_50_ (nM)	FC	EC_50_ (nM)	FC
Wild type HIV-1_IIIB_	0.11±0.02	1	201.89±34.11	1	9.39±1.22	1
FNC_P-21_ HIV-1_ IIIB_	80.82±8.14	735	123,690±18,321	613	12.29±0.24	1.31
3TC_P-21_ HIV-1_IIIB_	25.49±1.86	232	690,210±64,066	3419	13.13±2.43	1.40
HIV-1_LAI-M184V_	27.45±2.85	250	>800,000	>3963	8.21±1.30	0.87

***a***. Except for AZT, all data represent means ± standard deviation for three separate experiments. For AZT, the EC_50s_ represents means ± standard deviation for two separate experiments.

***b.*** FC, fold change, the ratio of EC_50(Mut)_/EC_50(WT)._

FNC showed good antiviral activity against clinical strains (Table 1). The EC_50_ values of FNC against HIV-1_KM018_, HIV-1_TC-1_ and HIV-1_WAN T69N_ were 6.92, 0.34 and 0.45 nM, respectively. Though FNC was less potent on clinical strains compared to lab-adaptive strains, the suppressive effects of FNC were 49 and 192 fold stronger than 3TC respectively. FNC was also inhibitory to HIV-2_ROD_ and HIV-2_CBL-20_ *in vitro* with EC_50_ of 0.018 and 0.025 nM respectively. These values were 12774 to 14259 fold lower than 3TC.

FNC and 3TC were both sensitive to NRTIs-resistant strain HIV-1_74V_, PIs-resistant strains HIV-1_L10R/M46I/L63P/V82T/I84V_ and HIV-1_RF V82F/184V_, and FIs-resistant strain pNL4-3_ gp41 (36G) V38A/N42T_ (Table 1). The EC_50_ values of FNC against these resistant strains were 0.11, 0.14, 0.37 and 0.36 nM respectively. These values were 1072 to 2968 fold lower than 3TC.

The EC_50_s of FNC and 3TC against HIV-1 in C8166 were unaffected by human serum in activity assay for 50% human serum (data not shown).

### Mechanism of anti-HIV activity of FNC

**Figure 2:**
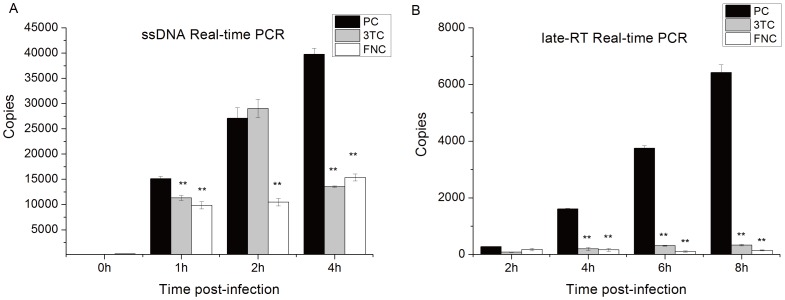
Mechanisim of action of FNC. Quantification of HIV-1 DNA species. ssDNA (**A**) and late-RT (**B**). All experiments were conducted in the presence or absence of 2nM FNC and 2 µM 3TC. Each group was tested in triplicates and data are presented as mean ± standard deviations (SD).

To elucidate the mode of action of FNC on reverse transcription of HIV, we detected unintegrated HIV cDNA (ssDNA and late-RT) using real-time quantitative PCR (Figure 2). ssDNA is an initial product of viral DNA and late-RT is the final product of reverse transcription [29]. C8166 cells were infected with HIV-1_IIIB_ in the presence or absence of FNC and 3TC. FNC at 2 nM significantly reduced the levels of ssDNA at 1 h, 2 h and 4 h post-infection(Figure 2A). It also significantly reduced late RT level in HIV-1 infected C8166 cells at 4 h,6 h and 8 h post-infection (Figure 2B) with a statistically significant difference between cells with and without FNC treatment (*P*<0.01). Similar results were observed with 2 µM 3TC and indicate that FNC and 3TC exert anti-HIV activity by blocking the whole reverse transcription process.

### Susceptibility of FNC and 3TC to resistant virus

After a serial passage of increasing concentrations of FNC or 3TC for 21 passages (70 days), it was apparent that viral variants were capable of growing in the presence of 3TC and FNC at concentration 64 fold higher than the initial value. The breakthrough viruses were obtained at P-5 (1 nM FNC and 2 µM 3TC), P-11 (4 nM FNC and 8 µM 3TC), P-16 (8 nM FNC and 16 µM 3TC) and P-21 (32 nM FNC and 64µM 3TC). Then the viruses were harvested and aliquoted in small vials. The antiviral activity against the virus at P-21 was assessed by phenotypic resistance assay. As shown in Table 2, the EC_50_s of FNC on FNC_P-21_ and 3TC_P-21_ HIV-1_IIIB_ were 80.82 and 25.49 nM respectively, which were 735 and 232 fold higher compared to wild-type HIV-1_IIIB_, respectively. Compared to HIV-1_IIIB_, the sensitivity of 3TC declined more significantly with the EC_50s_ increased for 613 and 3419 fold for FNC_P-21_ and 3TC_P-21_ HIV-1_IIIB_ respectively. HIV-1_LAV-M184V_ was used as a control in this assay since it is a highly resistant strain to 3TC. FNC showed cross-resistance to it with EC_50_ of 27.45 nM while 3TC could not inhibit the replication of HIV-1_LAV-M184V_ with concentration higher than 800 µM.

### Combination profiles of FNC with other drugs

The anti-HIV-1 activity of FNC was evaluated in two-drug combination studies with 6 FDA-approved drugs representing six types of antiretroviral therapy (ART) Drugs, namely AZT (NRTI), 3TC(NRTI), NVP (NNRTI), T-20(FI), RAL (INI) and IDV (PI). The CI was calculated by Calcusyn 2.1 software. Additivity, synergy, and antagonism were defined as detailed in Materials and Methods. Since low EC_50_s determined in anti-HIV activity assay, the dosages of FNC used in this assay were only 1000 or 500 fold lower than other tested drugs. The result demonstrated that all tested drugs showed synergism with FNC at EC_50_ on both HIV-1_IIIB_ infected C8166 and HIV-1_TC-1_ infected PBMC (Table 3).

**Table 3:** Effect of *in vitro* antiviral activity of FNC in combination with approved antiretroviral drugs in C8166.

Drugs	HIV-1_TC-1_/PBMC			HIV-1_IIIB_/C8166		
	Ritor	CI	Description	Ritor	CI	Description
FNC+AZT	1∶1000	0.44±0.20	synergism	1∶1000	0.60±0.17^a^	synergism
FNC+3TC	1∶1000	0.73±0.13	moderate synergism	1∶1000	0.61±0.12	synergism
FNC+NVP	1∶500	0.84±0.01	moderate synergism	1∶1000	0.64±0.22^a^	synergism
FNC+T-20	1∶1000	0.28±0.01	strong synergism	1∶1000	0.69±0.07^a^	synergism
FNC+RAL	1∶500	0.82±0.04	moderate synergism	1∶1000	0.65±0.01	synergism
FNC+IDV	1∶1000	0.50±0.15	synergism	1∶1000	0.52±0.13^a^	synergism

***a***. Data represent means ± standard deviation for three separate experiments. For other data represents means ± standard deviation for two separate experiments.

### Genotypic resistance analyze of FNC and 3TC from *in vitro* selection

To determine which amino acids are changed during in vitro selection, full RT genes (1680 bp) of both FNC- and 3TC-resistant strains were analyzed. Wild-type virus (HIV-1_IIIB_) was used as reference sequence. 146 positive clones of FNC (33 clones for P-5, 30 clones for P-11, 35 clones for P-16 and 48 clones for P-21) and 107 positive clones of 3TC (21 clones for P-5, 24 clones for P-11, both 31clones for P-16 and P-21) were picked and 3 mutation sites were observed based on T-vector clones sequencing (Table 4). We evaluated the frequencies of drug resistance-associated mutations in HIV-1 RT sequences obtained from these clones. The frequencies of M184I/V in FNC and 3TC induced variants presented obvious rising trend, but the frequencies of M184I and M184V were significantly different. The variants of FNC were gave preference to M184I, while those of 3TC preferred M184V. Among the variants of FNC, P-5, P-11 and P-16 had no M184V mutation and there was a 2.08% frequency of M184V in P-21. In 3TC- induced variants, a high M184V frequency of 45.83% emerged at P-11 and over 50% at P-16 and P-21. M184V/I was the highest resistant substitution for both FNC and 3TC which agreed with the result of phenotypic resistance assay. L214F showed high induced resistance correlation with FNC and the frequency of L214F ranged from 30.30% to 100% in P-5 to P-21 variants whereas similar. However, resistance correlation of L214F was not observed in the variants of 3TC. The frequencies of N519S located in the RNase H domain were also observed from 6.06% to 70.83% in P-5 to P-21 variants of FNC (Table 4).

**Table 4:** Genotypic patterns of HIV-1_IIIB_ selected by FNC and 3TC.

Mutation site	Mutation frequency(%)							
	FNC				3TC			
	P-5	P-11	P-16	P-21	P-5	P-11	P-16	P-21
M184I	3.03	66.67	97.14	95.83	4.76	45.83	25.80	47.83
M184V	0	0	0	2.08	0	45.83	70.97	52.17
L214F	30.30	76.67	94.29	100	33.33	8.33	3.23	26.09
N519S	6.06	54.05	74.29	70.83	4.76	4.17	3.23	0

### Computer modeling to predict the binding mode of FNC and 3TC to RT

**Figure 3:**
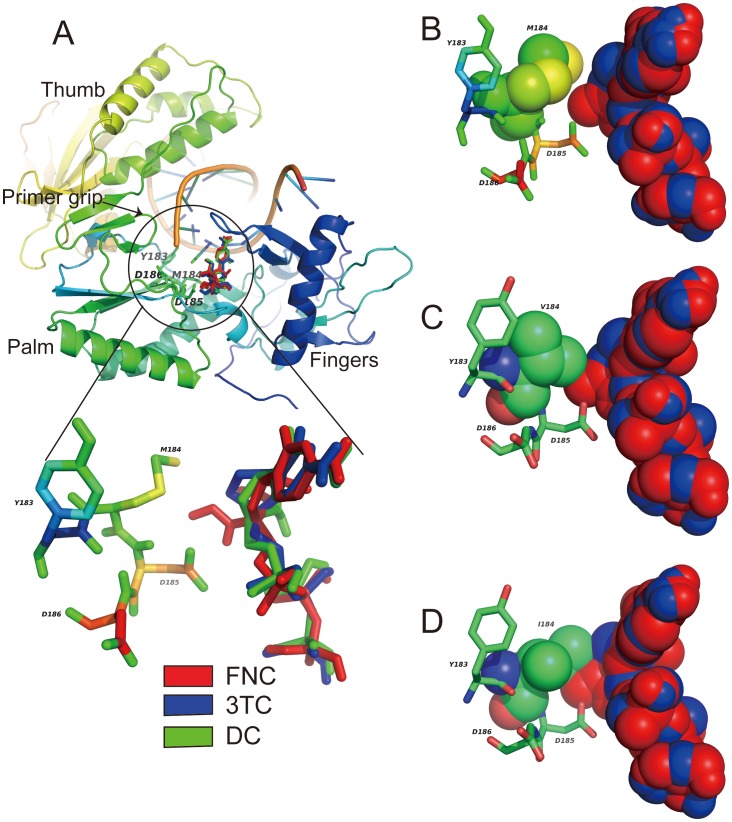
The binding of FNCTP and 3TCTP to the polymerase active site of RT. The binding mode of FNCTP and 3TCTP to RT of wild type and mutant forms (**A**). Top-panel and bottom-panel show the overview and close-up view of the binding mode of compounds FNC and 3TC, with DC as a control. Secondary structural elements were colored from blue (N-terminus) to red (C-terminus). The compounds FNCTP, 3TCTP and DCTP are colored with red, blue and green, respectively. The motif YMDD residues are shown as sticks. The spheres models of compounds FNCTP and 3TCTP interacting with residue184 of RT in wild-type M184 (**B**) and mutant forms V184 (**C**), I184 (**D**) are shown in the figure. The β-branched side chains of the residues V/I184 have steric hindrance with azido group of FNCTP and S atom at oxathiolane ring of 3TCTP.

As both AZT and the two compounds (FNC and 3TC) are NRTIs and share similar anti-HIV mechanism, we assume they have similar binding of the triphosphate moieties and base-pairing interactions with a modeled template overhang [30]. Recently, the complex structure of RT with AZT (PDB 3V4I) has been reported [31] and we used it as reference to predict the binding mode of the two compounds. The substrate of RT, DCTP, was used as a control and excellent consistency was observed between the two compounds and DCTP (Figure 3A), suggesting that our predictions are accurate.

**Figure 4:**
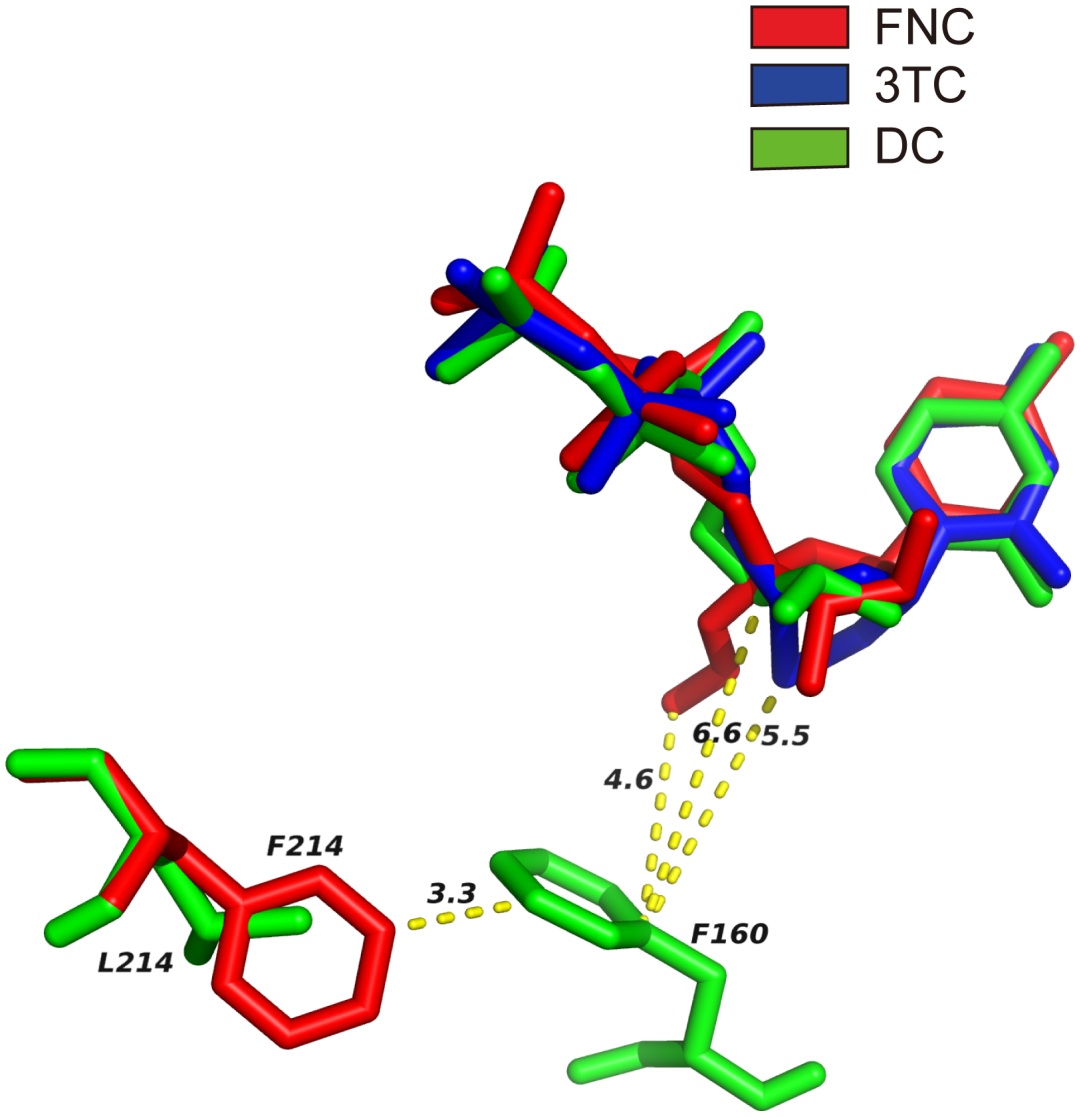
The interactions of FNC, 3TC and DC with residue 214 of RT. The interactions of FNC,3TC and DC with residues 214 and 160 of RT are shown. FNC, 3TC and DC are colored with red, blue and green, respectively. The residues 214,160 are shown as sticks. The distances between three compounds and the residues 214 and 160 are measured and shown by dashed yellow line.

Figure 3A presents the predicted pose of the two compounds and DC-TP binding with RT. FNCTP and 3TCTP occupy the polymerase active site of RT, and interact with the motif (YMDD) residues Y183, M184, D185, and D186. Compared with wild type M184 (Figure 3B), residues I/V184 have the β-branched side chains exerting steric hindrance on azido group of FNC and S atom at oxathiolane ring of 3TCTP (Figure 3C and D). The extent of steric hindrance differs between FNC and 3TC with the former causing greater interference in mutant M184I than M184V due to presence of the azido group (Figure 3C and D). This suggests mutant M184I is more resistant to FNC than mutant M184V. Since L214F showed higher induced resistance correlation with FNC but not 3TC, we examined the binding modes of FNC and 3TC with L214. Because of the unique azido group, FNC has hydrophobic interaction with residue F160 (4.6 Å), which is absent in 3TC due to the long distance to residue F160 (>5 Å). The emergence of mutation L214F will result in stronger hydrophobic interaction between residues F214 and F160, which will pull the F160 away from FNC. Thus, the mutation L214F disrupts the interaction between FNC and residue F160, it affects the binding of FNC but not 3TC to RT (Figure 4).

## Discussions

3TC is pivotal to all first-line ART regimens as it is a major NRTI recommended in the current HIV treatment guidelines. The compound is also effective against HBV [32]. However, 3TC resistance develops rapidly attributed to low genetic barrier and specific resistance towards 3TC are detected frequently [12], [33]. FTC is a NRTI structurally related to 3TC and shares comparable efficacy against HBV. However, it has a quite similar resistance profiles with 3TC [34]. AIDS requires long-term medication and necessitates the drug regimens to have low drug resistance and require less frequent administration.

In this study, we evaluated the cytotoxicity and antiviral activities of FNC against the HIV-1 lab-adaptive strains, clinical isolated strains from subtypes CRF_01AE and CRF07_BC, which are predominantly prevalent in China [35], [36], and RT resistant strains. We found that FNC showed higher cytotoxicity toward PBMC than C8166. FNC was highly effective on lab-adaptive strains and clinical isolated strains with EC_50_s lower than single-nanomolar. This indicates that FNC has better anti-HIV-1 activity than 3TC and FTC [34], [37], [38]. In mechanism study, FNC shown comparable activity in inhibiting reverse transcription products with the dosage only one-thousandth of 3TC (Figure 2). FNC was also highly sensitive to HIV-1_74V_, a NRTI resistant strain and HIV-1_WAN T69N_,a clinical resistant strain (Table 1). Although both FNC and 3TC showed high cross-resistance to HIV-1_LAI-M184V_, FNC still showed strong activity at nontoxic concentration (26.75 nM) whereas 3TC lost the activity (EC_50_>800 µM) (Table 3). Our results showed that the EC_50_ values of FNC were 2000 to 11000-fold lower compared to 3TC and those of FTC were 0.8 to 69-fold lower compared to 3TC [34]. Similarly, FNC showed stronger inhibition on HIV-1 and HIV-2 than 3TC and FTC, which implies a relatively low clinical doses of FNC may be sufficient for viral control and in turn trigger fewer side-effects.

Drug combination has been put in use to control AIDS since 1995 [39]. The adherence to ART has been strongly correlated with HIV viral suppression, increased survival rate and improved quality of life [40]. Since NRTIs play an important role in ART, it is necessary to evaluate their potential for synergy and antagonism. FNC was tested in the two-drug combination assays with representatives of six approved classes of HIV therapeutics and the dosage of FNC used were one-thousandth or one- five hundredth of the approved drugs. All tested drugs showed synergism with FNC (Table 3), suggesting that FNC had good combination prospects and potent inhibitory activity can be attained with low drug dosage. Our study will be useful for guiding the clinical medication.

Due to the absence of virological monitoring in routine clinical care and the use of ART drugs with low genetic barriers (such as 3TC and NNRTIs ), the emergence of HIV drug resistance mutations (DRMs) is challenging. To elucidate the resistance profiles of FNC and its difference from 3TC, we performed a 21 passages (70 day) selection in presence of FNC or 3TC. FNC- induced virus at P-21(FNC_P-21_ HIV-1_ IIIB_) was cross-resistant to 3TC (Table 2) showing 735 and 232 fold higher EC_50_ in comparison with wild-type HIV-1_IIIB_, respectively. The sensitivity of 3TC declined more significantly both to FNC_P-21_ and 3TC_P-21_ HIV-1_ IIIB_ with 613 to 3419 fold higher EC_50_ in comparison with HIV-1_IIIB_. HIV-1_LAI-M184V_ was used as control in this assay since it was a highly 3TC resistant strain. FNC showed cross-resistance to it with EC_50_ of 27.45 nM while 3TC could not inhibit the replication of HIV-1_LAI-M184V_ at concentration up to 800 µM. The results of the present study therefore suggest that FNC may be used as a replacement deoxycytidine analogue for 3TC in patients harboring the M184V mutation. FNC has been approved for clinical trials by China Food and Drug Administration (CFDA) in April 2013. Clinical studies will be necessary to assess its effectiveness *in vivo* when used as a replacement for 3TC in patients with 3TC resistance.

Our genotypic analysis revealed amino acid changes were associated with FNC resistance. The dominant mutation site associated with FNC resistance was M184I (from 3.03% at P-5 to 95.83% frequency at P-21) whereas M184V was only detected at P-21 with 2.08% frequency (Table 4). M184V was found in 3TC- induced viruses except for P-5 (45.83% frequency at P-11, 70.97% frequency at P-16 and 52.17% frequency at P-21). Since M184I was transient and replaced by M184V in 3TC-resistant patients [12], [41], this suggests FNC might have different mechanisms for 3TC resistance. In FNC- induced HIV, mutation frequencies of M184I at P-5 and P11 were 3.03% and 66.67%, respectively, whereas in 3TC- induced HIV,M184I frequency at P-5 was 4.76% and M184I/V frequency of at P-11 was 91.66%. This might imply FNC has higher genetic barrier than 3TC. The most frequent RT mutations selected by 3TC were M184V and K65R [14], [42] and it will be interesting to determine whether *in vitro* selection assay also contains the K65R substitution. In present study, no mutation of FNC or 3TC was found at K65R which is probably because of the short induction time. In addition, we identified another highly frequent mutation of FNC–L214F (from 30.30% at P-5 to 100% at P-21) which was polymorphic in viruses from both treated and untreated patients [43]. N519S is another common mutation in the RNase H domain with frequencies ranging from 6.06% to 70.83% in FNC- induced variants. Interestingly, N519S and L214F were found in 3TC-selected strains without obvious resistance correlation. Four additional amino acid changes (R461K, T468P, D471N and A508T) were also detected in the RNase H domain without obvious correlation (data not shown). The different drug resistance profiles between FNC and 3TC should be intriguing from a structure-activity relationship point of view.

Computer modeling was performed to predict the binding of FNC and 3TC to RT and in turn elucidate the higher level of resistance of M184I and M184V mutants for 3TC than FNC. It was reported that 3TC have the ribose ring replaced by an oxathiolane ring and the stereochemical form of 3TC used to treat HIV-1 infections is the opposite enantiomer relative to normal dNTPs. The introduction of a sulfur atom into the ribose ring and use of opposite enantiomer causes the sulfur-containing oxathiolane ring projects farther than the normal ribose ring, thus results in steric hindrance [30], [44]. In our study, we showed that single mutations at residue 184 of RT in HIV caused resistance to FNC and 3TC (Table 2 and 4). Compared with wild-type M184 (Figure 3B), residues I/V184 have the β-branched side chains which have steric hindrance with azido group of FNC and S atom at oxathiolane ring of 3TCTP (Figure 3C and D). This observation is consistent with the results of genotypic resistance analysis in our study, which showed that both mutations M184I and M184V emerged in the HIV-1IIIB strains selected by FNC and 3TC. The predicted binding model also explained the difference in steric hindrance between FNC and 3TC. The presence of azido group in FNC leads to stronger steric hindrance in mutant M184I than M184V (Figure 3C and D) whereas the structural interferences were comparable in mutant M184I and M184V for 3TC. This analysis suggests mutant M184I is more resistant to FNC than mutant M184V and explains the higher mutation frequency of M184I than M184V in the FNC-selected HIV-1IIIB strains. In order to explore how L214F mutation affects the binding of FNC to RT, we compare the binding modes of three compounds namely FNC, 3TC and DC (Figure 4). FNC has hydrophobic interaction with residue F160 (4.6 Å) by azido group, whereas 3TC and DC do not have this interaction because of long distance to residue F160 (>5 Å). The emergence of mutation L214F will result in stronger hydrophobic interaction between residues F214 and F160 which draw F160 away from FNC. This analysis is explanatory to the fact that the more FNC resistant viruses contain L214F. The N519S mutation also present a relevance to drug resistance, but it is located in RNase H domain, which is far away from the binding site of FNC. The distance between N519S and FNC reaches almost 50 Å. Therefore, it is difficult to explain the mutation according to the structure of FNC. R461K, T468P, D471N and A508T mutations were also detected in the RNase H domain, these mutations may be an adaptation in overall structure, which have synergistic effect in the drug resistance.

In conclusion, we present FNC, a new NRTI, which shows higher anti-HIV activities to different lab-strains, clinical-strains and resistant-strains than 3TC. Azvudine (FNC) is a promising drug to be used in therapy or served as replacement of 3TC for treating HIV-1 infection. FNC can be a new option for HIV-infected patients and further clinical tests on its effectiveness and tolerability should be done in the future.
